# Learning to teach by teaching your peers: exploring students’ needs for training in the undergraduate medical education curriculum

**DOI:** 10.1186/s12909-025-07022-z

**Published:** 2025-03-21

**Authors:** Sara George Sahba, Anna Bonnevier, Terese Stenfors, Ellinor Kenne

**Affiliations:** 1 Department of Physiology and Pharmacology, Division of Molecular Exercise Physiology, Karolinska Institutet, Biomedicum 5C Karolinska Institutet 171 77, Stockholm, Sweden; 2 Department of Learning, Informatics, Management and Ethics (LIME), Division for Learning, Karolinska Institutet, Stockholm, Sweden

**Keywords:** Medical education, Peer assisted learning, Peer teachers, Peer learners, Teacher training, Curriculum development, Medical students, Qualitative research, Focus groups

## Abstract

**Background:**

Different forms of peer teaching are commonly used in medical curricula. Peer assisted learning (PAL) creates an opportunity for medical students to practice teaching and communication skills. However, more data is needed to identify how peer teachers undertake their teaching assignment and what support they need during medical school as well as to feel comfortable to teach as practicing physicians. Therefore, this study aimed to investigate medical students’ experiences of peer teaching with focus on what obstacles students meet, what support they lack, and how this support, according to students, should be designed.

**Methods:**

A qualitative interview study was performed, collecting data through semi-structured focus groups and individual interviews with peer teachers (PTs) and peer learners (PLs). Data was analyzed with thematic analysis.

**Results:**

The qualitative thematic analysis was based on the research questions and resulted in six themes: becoming a PT or not; experiences of received support during peer teaching; experiences of teaching– developing as a teacher; experiences of teaching– finding support for the teaching role; including PAL as part of the curriculum– desired structure; and including PAL as part of the curriculum– desired content and teaching modalities.

**Conclusion:**

This study supports the idea that, in addition to enhanced subject specific knowledge, PTs can develop qualities useful for the educational assignment of medical doctors. More importantly, we add novel knowledge by showing that there is often insufficient formal training in the medical undergraduate curriculum for students to be sufficiently prepared for this task. To reach learning outcomes in teaching and communication, students want a training program to include a theoretical base and to learn by contextualized practical experiences with feedback. Inclusion of mandatory peer teaching in the medical curriculum should consider timing, the subject area that is taught and proper training for the peer teachers.

**Supplementary Information:**

The online version contains supplementary material available at 10.1186/s12909-025-07022-z.

## Background

Teaching skills are internationally agreed upon as a requirement of medical students upon graduation [1, 2]. One way to train teaching skills is through peer teaching and today many medical curricula contain some form of peer teaching [3]. Peer teaching can be designed as senior students teaching junior students, where the senior student is the peer teacher (PT) and the junior (i.e., the less experienced) student is the peer learner (PL). Peer teaching has been used in both preclinical and clinical learning situations [4] and is a successful teaching and learning strategy which may improve students’ academic performance. The social and cognitive congruence between PTs and PLs are major contributing factors to the success of peer teaching programs [5]. PTs’ recent exposure to the material as well as the possibility to advise on how to cope with medical school and understand possible struggles [6] are other factors that contribute to peer teaching being a positive experience for the PL. Although there are few studies systematically investigating what PTs learn from teaching, evidence suggests that peer teaching offers learning potential for PTs and supports them to become better doctors, better learners, and better supervisors during residency [7, 8]. A peer teaching program can provide realistic training experiences in teaching and increasing interest in an academic career [9]. Additionally, peer teaching could improve PTs’ theoretical knowledge, presentation and teaching skills, as well as teamwork and leadership skills [10] all important for a practicing physician.

Hence, including peer teaching for all medical students could support their future role as teaching physicians, and incorporation of near-peer teaching in the mandatory curriculum benefits both PLs and PTs [10]. To ensure that peer teaching maintains high-quality and supports learning, PTs need training, supervision, and feedback on performance [11]. Despite this, previous studies have shown that teacher training programs for medical students are few, often non-mandatory and targets students in later years [3, 12]. Providing adequate training and support to student teachers could enhance the learning experience for both PTs and PLs [8]. However, to facilitate implementation of successful programs more knowledge is needed on what type of support and training medical students think is necessary.

This study aimed to investigate medical students’ experiences of peer teaching, focusing on how PTs approach their teaching assignment, what support they need, and how this support, according to the students, should be designed. Based on this, together with findings from previous research, it is possible to delineate how to develop peer teaching programs, in undergraduate medical curricula with special focus on how to support and prepare PTs for the teaching assignment.

## Methods

### Study design

To investigate students’ experiences of peer teaching, a qualitative study design was chosen. Data was collected over a period of three months (February to April 2022) through semi-structured focus group interviews with PTs (students with peer teaching experience) and PLs (students without peer teaching experience) from the Study program of Medicine at Karolinska Institutet (KI), Stockholm, Sweden. We included PLs in the study as we wished to explore, for example, barriers to become a PT and their thoughts on teacher training for students.

### Setting

At the time of this study, the Swedish study program in Medicine was 5.5 years (330.0 ECTS). Since the interviews were completed, the Swedish medical schools have implemented a six-year program (360.0 ECTS). In both programs at KI, peer teaching is mainly used in the first three semesters in the basic science subjects anatomy, histology and physiology. PTs are recruited in the semester after they have completed each respective subject.

### Participants

Students were invited either by direct contact (*n* = 40) by email, or by a request distributed using the learning management system reaching approximately 300 students. Inclusion criteria for PTs were that they had fulfilled at least one PT-assignment in anatomy, histology and/or physiology at KI. Inclusion criteria for PLs were that they had partaken as a student in peer teaching. Separate group interviews were held with PTs (*n* = 16) and PLs (*n* = 9) respectively. Some participants had graduated from the medical program at the time of study. The interviews with former students (*n* = 5) were, for scheduling reasons, done individually or in pairs.

### Data collection

The interview guides were constructed by the research team (Supplementary materials 1). They consisted of a mixture of open questions e.g., PTs’ experiences of the impact peer teaching might have on their professional development, and what motivated them to take the assignment. PLs were asked e.g., about their experiences of peer teaching as an educational method and why they chose not to take on a PT-assignment. The guides also had more focused questions related to the research questions, such as perceived needs for educational or other support and how potential training should be designed. The interviews were open and relatively free for explorative discussion among the participants.

All interviews, both group and individual, were conducted through Zoom and moderated by one investigator (SGS), a female senior medical student. The participants were informed that the transcripts would be anonymized. The interviews were conducted and parallelly analyzed until the point of information power [13] was considered high.

### Data processing and analysis

All interviews were recorded and transcribed verbatim by the same member of the research team (SGS). All data were pseudonymized during transcription. Recordings and transcripts were saved on a secure server, to which only the research team had access. Once the transcripts had been finished, the video recordings were deleted, and only the audio files were stored. Interviews were performed in Swedish, and citations were translated to English by the research team.

The interviews were analyzed with qualitative thematic analysis, with the research questions as a starting point and focus on finding shared meanings across the data set. The analysis was made by the research group first individually and then together using the six-phase framework of Clarke and Braun [14], an iterative and collaborative process moving between data, codes, and themes several times both individually and collectively, before reaching a final version. Codes were extracted from data, creating sub-themes and themes. Three of the co-authors (SGS, EK and AB) participated in all the steps of the analysis and read all the transcripts whilst one of the co-authors (TS) read a selection of the transcripts and commented on the preliminary results throughout the process. We acknowledged the possibility of various ways of interpreting the data between the researchers and saw our subjectivity as a resource during reading and processing of the interviews and in identification of analyzed units of meaning.

Preliminary findings were presented at AMEE 2023 and the feedback received supported the credibility of the study and helped us strengthen applicability and resonance. Selected aspects of interview data from PTs were subsequently included in the forthcoming manuscript *Learning outputs for peer teachers in undergraduate medical education* (Skjærseth et al.) focusing on learning outputs for peer teachers in undergraduate medical education in different contexts, as well as the prerequisites that lead to these outputs.

### Research team and reflexivity

The research team consisted of individuals with a strong background in qualitative research, as well as faculty with expertise in education and deep understanding of the context of the study. SGS had some personal experience of working as a PT in histology and as a PL as a medical student. She had limited training in research methodology before the study commenced but was supported by the co-authors. At the time of conducting the study SGS was an 8th semester medical student performing this study as her master’s thesis. EK is a PhD and teacher in the medical program with experience from organizing peer teaching in physiology. She had personal experience in teaching together with some of the interviewed PTs, but was blinded of their identities throughout the project. Their deep understanding of the peer teaching context in which the study took place and the medical program helped contextualize the data and facilitated the analysis. AB and TS are medical education researchers with expertise in qualitative research, both have previously conducted research in the area of peer teaching but none have had an active role in the study program where this study took place, this helped increase confirmability and transferability, balance the analysis in terms of focusing on the data at hand and comparing and contrasting with previous research.

## Results

### Study population and interview data

A total number of nine group interviews, lasting 50 to 75 min, were conducted. The three individual interviews lasted 20 to 40 min. An overview of the respondents’ characteristics is presented in Table 1. Peer teachers had experience in teaching anatomy, histology and physiology for students in the second and third semester in the medical program as well as in other health profession programs. Teaching mostly consisted of practical small group sessions, with 4–12 students per group, focusing on using the microscope, dissections and physiology labs. The peer teacher group was heterogenous with regards to how long they had been active ranging from one to eight semesters. The thematic analysis resulted in a total number of six themes related to the research questions, with three sub-themes each. Themes and subthemes, as well as examples of corresponding quotes, are presented in Table 2.

**Table 1:** Overview of respondents’ characteristics

	Peer teachers	Peer learners
Gender	8 female, 11 male	6 female, 5 male
Age	23–31 years old	21–40 years old
Semester in study program/Number of graduates	Semester 5–10 3 graduates	Semester 4–8 2 graduates

**Table 2:** Result of thematic analysis presented in themes with representative quotes from peer teachers and peer learners. Interviewees 1–25 were current students in the medical program, and interviewees 26–30 were graduates.

Themes/Subthemes	Peer teacher (PT) quotes	Peer learner (PL) quotes
**Theme: Becoming a PT or not**		
Intrinsic motivation	It’s simply fun to have a reason to continue with physiology. It’s very fun and fun labs and to deepen my knowledge a little. PT5 It’s just that feeling of paying forward to a person that doesn’t know something that I know, I think that’s satisfying. PT18 It’s a lot of fun, you get a lot of energy, it’s social too, both with other tutors and meeting students from younger cohorts. PT19	When I think about it now, I probably would have wanted to do it [be a PT], because then you keep your own knowledge fresh in mind. PL12 I think that it strongly contributes to building character and self-confidence. PL24 It felt like a lot of pressure (…) you don’t want to sign up to be a tutor and then be one of the tutors that doesn’t help (…) so I felt like there would be others that could do a better job than me, so then it was just as good to refrain. PL25
Extrinsic motivation	I think it [peer teaching] will be useful in the future, the knowledge you get, but also that the assignment itself can be meritorious. PT7 But also, that you get paid for it. It has been a driving force. PT10 This shows that I am driven and ambitious, because there is no other way to stand out except for these kinds of activities. PT19	I think one of the driving factors is the economical, because it can be considered as a side job. So, I think that financial compensation is important. PL11 They ask for pedagogical or other assignments of trust in residency applications. PL26
Administrative circumstances: time, logistics and availability	I was only an anatomy tutor and I think I applied for the super-tutor position, but I didn’t get it, so it ended there. PT2 Being able to match the schedule with your own hospital [schedule] and get to [campus], was a bit tricky (…) then there was no point in continuing. PT3 I thought it was difficult to prioritize it over my own studies. PT14	I think I thought about it, but I decided not to do it, partly because I have quite a lot of other engagements. PL12 For me the time [it takes] made it impossible. I travel back and forth which takes 20 h a week, and it would have been impossible to be there to engage. Otherwise, I would have been interested, but there was no time. PL13 I definitely would have had that interest, mainly in chemistry. Not so much in anatomy, it’s not my thing. PL22
**Theme: Experiences of received support during peer teaching**		
Training in pedagogy and teaching methods	The introduction was very short and superficial, and mainly dealt with how to support the students in the anatomy department [the dissection room] rather than in their learning, since it can be a bit stressful. That was useful, I had a use of it. PT2 It is really lacking [pedagogical training]. It’s again that it varies so much (…) where everyone does it their own way and everyone thinks it’s good. But no one really knows. Maybe I am missing something, but it would be interesting in any case with some actual evidence-based pedagogy teaching, how one should actually do it. PT4 We didn’t get any training or lectures explaining how it should be, i.e., to teach others. We should get some additional training in just that– teaching– some pedagogical education. PT18	
Clear instructions and explanation of the task	One thing that has been very good with physiology (…) is that we had pre-labs for the slightly more difficult labs. Because obviously there are a thousand different ways to go when describing concepts and such. Not least in physiology, so then they have this good thing that we get together and talk about what key concepts we have and how we should do it, which are the best ways to go. Then we sit down and discuss before, it’s pretty good, because then we come to an agreement. PT6 There was an instruction manual or template that you followed (…) there I thought it was quite clear and structured what you had to do, which steps. But the teaching itself and the pedagogy part itself was a little free. PT14 So, you have like an hour the day before so you go through the lab with the responsible teachers and can ask questions yourself if there is something you think was difficult and where you go through all the steps that you will do the next day. PT17	
Support and dialogue with teachers or other PTs	And then, we usually stayed a bit afterwards to talk: “today I mentioned this, it was a bit unwise and was very confusing”, so you talk to each other and try to convey what you have taken with you from the session. PT4 And then there was very good support from super-tutors and teacher X who were there. I thought it was fun and ran very smoothly. PT15 We got quite a lot of support from teacher Y, afterwards, during the lab and afterwards. You get feedback then. And you can see how the faculty, teacher Y and the other physiology teachers, answer questions… PT8	
**Theme: Experiences of teaching– developing as a teacher**		
You do not have to know everything	I had some idea that you need to know everything and be able to answer everything correctly and, that the whole world falls apart if you can’t answer a question, but you have learned little by little that this isn’t really the case. PT5 I thought that I would be surer of my answers the more senior I became, and the more semesters I had taught, so that I could answer more quickly and more directly. But I was very surprised that it was the opposite. It’s like, the more you know about a subject, the more semesters you have behind you, the more you think when giving an answer. PT8 …you become rather flexible and accommodating to the fact that it can be any type of questions and maybe those that do not pertain to the topic. Then you will see if you can answer them or not. PT28	
Adapting to students’ needs	It is difficult to teach so that it fits a larger group. I only know how it works for me and how I have learned. You noticed, it shouldn’t come as a shock and maybe it wasn’t a shock, but specifically to adapt the teaching to everyone, it was probably like, it was much more complicated than perhaps what I had imagined. PT2 It is incredibly individual what the students want help with. Some students just want to sit back and have things explained, while others want, have smaller issues they want you to add to. You really must adapt depending on which student you have in front of you. PT17 But it’s funny how many of the students who usually have the same questions. Sometimes it’s like 6 just said: wow, what was that question? But something I did not expect was that, if you are teaching the same module, it is quite similar what questions [students ask] and the students’ level of knowledge. I didn’t think so, I thought it would vary a lot all the time. PT7	
Peer teaching is to facilitate PLs’ learning	I had some idea that you would stand actively and give a lecture or something or tell the students how it works. But you noticed quite quickly that they solve everything themselves, you are only there as a support. PT5 But also, to teach how to arrive at thinking, the actual thought process when you don’t know the answer right away. That like the whole, the navigation bit and so on: “ok what surrounding structures can we use to move forward”. PT14 I have something that I do now as a supervisor, which I wouldn’t have done otherwise I think [if I didn’t teach]. I often try to get the students to express what they want to do and what they want to learn, and I try to discuss with the students so that they themselves can draw conclusions and help them along the way when they need it. So that it’s not like cathedral lectures in the clinic, but that they get to reason more independently. I think that’s a big difference. PT29	
**Theme: Experiences of teaching– finding support for the teaching role**		
Using PLs’ feedback to support your teaching	You still got feedback from the students you had. So that you could develop in that area. PT1 I think you notice partly if they keep up and are engaged and partly if it feels like you have some good communication during the session. If the students are not satisfied, you will notice it quite quickly because they will not be as engaged and will ask fewer questions. PT5 You realize the importance of feedback later. Right now, I always try to ask the students “how did you think that this went… I would really like to know what you think. Did it go well? Did you get your questions answered? Did you come out with more knowledge than you came in with?” PT8	
Using teachers or peers as role models	I think I just followed the model that existed. Just like previous tutors had done for us. And as everyone else basically did too. PT1 And it’s also a lot, you hear what other people do, I might pass by and hear 6 and 7 talking about something in a smart way or mention something, then I think that it was smart, I’ll talk about it. PT4 You see how your own supervisors do when you work, and you take after their style of supervision. PT30	
Using your own experience of being a student	I only had myself and my closest friends, their experiences to work from. Which means that when we encounter students that were not comfortable with the situation and had principally completely different experiences, I had to adapt my pedagogical approach, but I don’t feel that I was prepared for that. PT2 I structured the session so that I taught the students how to look at a sample to quickly learn what organ it is. Because that’s what’s important in the examination… And then I said that if you want to know more details, ask me and we’ll go through it. But when I was in semesters 1 and 2, it didn’t interest me. I wanted to know, what did I have to know to pass the exam… PT3 I would have wanted to make it more fun for the students. Maybe added, in different contexts, more surgery. I had a tutor who always talked about surgical procedures and that made you remember better. PT16	
Informal extra-curricular training opportunities	I think about what I learned from being a student mentor more than being a histology tutor. It feels like you are more important to the new student in that role. PT3	I have some experience in health care where I supervised colleagues. PL11 I think I’ll be adequately prepared, not necessarily by pedagogical knowledge I’ve gained from medical school, but I work with kids in […], where I’ve done leadership courses and teaching courses. PL12 I had a pretty long experience in supervision before I started the medical program so I feel pretty comfortable. PL13
**Theme: Including PAL as part of the curriculum– desired structure**		
Mandatory or not	I don’t think that [PAL] should be mandatory, it already is in a way with patients off and on: that you explain things, but I don’t think that it would have been good to implement something mandatory. PT1 I think so [PAL should be mandatory]. Because it’s an important part of the medical profession, to have at least some knowledge of how pedagogy works. And for one’s own learning, I think. PT9 Making [teaching] mandatory would lead to lower quality because a lot of people would not want to do it. PT19	I don’t think that it should be mandatory… I think that it becomes forced when it’s mandatory. When you’re not really engaged, or don’t think that PAL is motivated, which I think is more likely when it is mandatory. PL12 But I think it would have been good, because I think that it’s often the same type of people within the class that take on the leader role, or the role where you teach others. It’s probably good that everyone in the class gets to practice taking on this role. I think it contributes to building character and self-confidence. PL24 I think it’s reasonable to have some type of teaching assignment during the medical program considering that it’s practically guaranteed that if you work clinically, or in research, will have teaching assignments. PL27
Allocated time and timing	Theoretically, it sounds great to have the opportunity to develop your knowledge, but at the same time I think that if you are like 3rd or 4th semester, you already have the tutoring thing alongside your full-time studies, you have the upcoming pre-clinical exam (…) I think we medical students are very much like this: is this something that I will benefit from now or possibly in the near future, or in the upcoming exam. But if it is just a deep dive into something that might come up two years from now and be forgotten anyway, not many students will show up. PT2. It could be anything from a full course to an hour and I think it should be really short. PT4 I think it should be a part of the professional development track… Since it’s a part of being a professional in the profession that we have chosen, to be a good supervisor. PT14	I think that it would have fit in semester 11 [last semester]… it would be good to have some pedagogy then since you will soon work as a resident and then you will have to supervise. All medical students appreciate a good supervisor, so it’s right. It can make a big difference if you have some pedagogical experience of teaching. PL12 And if you teach things too early, there is a risk that they won’t be used. PL21 … it sounds like a huge project (…) but maybe it could be like a thread throughout the curriculum. PL23
Quality assurance	If [teaching] would be mandatory (…), it would have to be assessed at the same time too because otherwise those that don’t want to do it will do a poor job and then the students [taught] will be affected. PT7 Maybe quality varies between the tutors because we are not assessed or evaluated in the same way as we assess the students. PT16 If you’re not interested in it [teaching] and not ready for it at the time of your training, it might not be good for those who are being taught. PT30	There are lots of conceptual things that has a lot of detailed knowledge in it. And in order to be able to teach it in a good way you really must know it well, you really have to be able to explain it well. There you can really fail as a student if it doesn’t work well […] And a thing like that may have to be explained by someone who understands the big picture compared to someone who has recently learnt it at the same level as we are supposed to learn it. PL13 That it’s clear for everyone, that it’s clear and professionally structured. PL21 I also spontaneously think that quality assurance is incredibly important here, it’s sort of central. You would not want to learn something that is incorrect and take it further and risk that it travels further down the line and that it is not caught and just continues. Quality assurance is vital. PL22
**Theme: Including PAL as part of the curriculum– desired content and teaching modalities**		
Evidence-based educational theory	It would have been rewarding for oneself to know how you really should do it and if your methods are evidence based. PT5 There must be many evidence based pedagogical methods out there, there are. So that would be great. PT6 If everybody would get a basic review of the science of how people learn best [before starting teaching], the starting point would have been more equal. PT14	You should get one or a few basic lectures about learning styles. Because people learn in different ways and they say that some learn by hearing, some learn by seeing and some learn by doing. PL12 Evidence based. PL20 …how people learn best, and how to get what they learn, facts, to stick… That the pedagogy also is evidence-based. It’s not only research but also pedagogy that should be evidence-based. PL22
Teaching skills	I agree that communication is important. Presentation techniques are important. Pedagogical skills are important. They all go hand in hand. PT8	…some kind of mini seminars where you can discuss, with someone knowledgeable, how to handle tricky questions, how to answer them. How do you turn things around, practice and maybe train one’s own insecurity away, if one has that. PL13 … because it’s so important. Supervision and teaching… is a lot about communication and for doctors it’s extremely important that you can communicate with patients and colleagues. PL25 Different methods you can use to try to find out knowledge or try to connect theoretical and practical knowledge. And if it’s about retelling or trying to simplify. Yes, different tools you can make use of. PL26
Contextualized practical experience with feedback	… interactive in small groups where you get to try because it’s one thing to understand the concepts, but it’s another thing to try it and then to get feedback that “you missed this now”, because you can think that you perform in another way than you actually do. PT6 It would of course have been better with some more hands-on training. Both theory and to maybe shadow someone once or twice to get to know their thoughts about what went well and not. PT8 It must be an activity that’s practically applicable and really contributes to learning. PT10	… that you get feedback maybe. Like some kind of sit-in, like we have with patients, but that you do it as a supervisor instead. PL12 …it takes time to feel comfortable [teaching]. Just like with patients, it compares really well to that I think. Getting patients to talk and to teach them is something many of us are not used to. It takes practice to become good at it. PL23 …that you [the student] gets feedback; “you did this well, and you could have thought about this”. PL25

### Becoming a PT or not

Students chose to become PTs for different, intrinsic or extrinsic, reasons. Personal gain was, according to PTs, an intrinsic factor resulting from the PT assignment and included having fun at the job, participating in a social activity as well as the positive feeling of doing an altruistic deed by helping junior students learn. Many interviewees became PTs for the opportunity to rehearse and develop their subject knowledge, which was also recognized by many PLs as a possible positive learning outcome of peer teaching. Increased teaching skills were expected by both PTs and PLs and were considered especially important for the future medical profession. PLs also believed that being a PT would lead to the development of generic professional competences such as self-confidence.

As PTs get paid for their work, the financial benefit was an extrinsic motivator that contributed to the decision to take on a PT-assignment. This was also recognized by PLs as a motivating factor. As the grading system in Swedish medical programs is pass/fail only, some students saw the PT-assignment as a professional merit and an opportunity to show motivation and high achievement to future employers.

A common barrier for becoming, or continuing as a PT, was time and logistics. Students had to skip lectures and lost individual study time to work as a PT. During later semesters the logistics of combining clinical rotations with peer teaching on campus was a major difficulty. Students also described personal and external factors that prevented them from becoming a PT. For example, lacking the confidence to perform as a PT was categorized as a personal limitation by PLs. That PT-assignments were only offered in a few subjects and that the current PT-program in anatomy only offers advancement for a limited number of PTs were other external limitations.

### Experiences of received support during peer teaching

According to the students interviewed, there was no formal teacher training included in the medical curriculum. When PTs were asked what specific type of training they had received as preparation for their peer teaching assignment, it was evident that a majority experienced that they had not received any formal training in pedagogy or teaching methods, nor any subject specific training prior to their PT-assignment. Even though the PTs in anatomy and physiology mentioned introductory information meetings, they still answered that they did not get training to become a PT, indicating that the meetings were not perceived as such. However, the information meetings contained practical information, instructions on what subject areas to cover, and some pedagogical advice, like letting the students think for themselves and keeping them active. PTs in anatomy also talked about receiving information on how to deal with PLs who might be upset by the dissection situation. PTs found these preparatory meetings short, but useful. An informal aspect of support that PTs mentioned was the possibility for dialogue with teachers and other PTs which was perceived as very useful by the PTs.

### Experiences of teaching– developing as a teacher

Many of the interviewed PTs emphasized that a surprising discovery from teaching was that as a teacher you do not have to know everything and that there is an acceptance among learners that sometimes you need to look things up or find things out together. Many of the PTs with extensive experience of teaching reflected on how teaching had helped them develop a more professional attitude. They were able to identify strengths and weaknesses in their professional approach, for example being able to assess and identify personal limitations in knowledge and taking responsibility for reducing the risk of providing PLs with inaccurate information.

The teaching experience also led to PTs obtaining a more student-centered approach to learning, e.g., recognizing the importance of teaching at an appropriate level and adapting the session to each student’s performance. This was considered as a challenge by some PTs while others thought it was interesting to learn that many students end up having the same questions or experience the same difficulties with a subject. Another student-centered aspect was that PTs developed the perception that their main role as teachers was to be supportive and facilitate the PLs’ learning process, rather than lecturing.

### Experiences of teaching– finding support for the teaching role

Despite the experienced lack of formal support for PTs, the interviewees described several strategies used to manage and improve in their teaching role. For example, PTs used the feedback they received from PLs. They received feedback primarily by asking the PLs what they thought about the session and wanted to learn more about. Other forms of support for the teaching role expressed by PTs were to observe other more experienced PTs or clinical supervisors, copying appreciated behaviors and methods, or in some cases just replicating an existing model. PTs also used their own experiences of being a student or utilized written or oral feedback given by PLs during or after teaching to develop their teaching.

Since teacher training was not part of the mandatory curricula, both PTs and PLs described different informal or voluntary training possibilities (beyond being a PT in physiology, histology, or anatomy) to practice teaching skills during the medical program. This included activities such as presenting assignments to peers, arranging mock OSCEs as training exercises for junior students or volunteering as a mentor for new students. Besides the opportunities within the medical program, some students described extracurricular training as an option to engage in a teaching role. These included personal engagements such as side jobs, e.g. training kids in sports, or other experiences before medical school. However, students emphasized that these, often voluntary, training opportunities did not give equal possibilities to develop teaching skills for all students.

### Including PAL as part of the curriculum– desired structure

Themes about including PAL as part of the curriculum include both students’ suggestions on what such training could contain in terms of content and how it should be structured and designed.

There were mixed opinions among students, both PTs and PLs, towards including mandatory peer teaching and teacher training in the curriculum. Several students expressed that PLs did not teach simply because they did not want to. Students were worried that including mandatory teaching for all medical students would lead to decreased educational quality for the PLs. Apprehensions were that PTs would be demotivated, unengaged or that they would teach something that was incorrect. At the same time other students indicated that it would be a chance for students that normally would not take on that role to gain teaching experience since it is an important part of the medical profession.

Students were often concerned with how a course in pedagogy, teacher training or additional peer teaching possibilities should be possible to implement in an overloaded curriculum. There was unity in the belief that if implemented, such a program should not be too excessive. Other opinions were that the course could be taught continuously during the program as part of for example a professional development track, and that timing was important. Interviewees highlighted the need for quality assurance of peer teaching to minimize the risk of negative effects on PLs’ learning. Suggestions for making the peer teaching assignment more attractive to students and avoiding negative effects on learners were that the PTs should be able to choose the subject area in which they taught, and/ or that the teaching activity would be included as a mandatory activity in a specific course and assessed by a faculty member. Clear guidelines for the PT-assignment were also important.

### Including PAL as part of the curriculum– desired content and teaching modalities

Analysis of the interviews clearly showed that many students believed it would be useful to learn about and practice teaching during the medical program. Both PTs and PLs wanted to learn evidence-based teaching methods and/or learning theories. Other aspects of content to include were presentation techniques, how to adapt to students’ different needs, methods to engage and motivate students, how to handle difficult questions in the subject being taught as well as connecting theoretical knowledge to clinical practice. It was also important to students that a training program included communication skills, including communication with students as a supervisor.

It was very important to students that a potential training program included practical elements and the opportunity to practice with feedback from supervisors or teachers. The practical exercises should be set in fitting, realistic contexts, such as supervising junior students during clinical semesters to prepare PTs for supervision as junior physicians. Teaching modalities mentioned by the interviewed students included e.g. role play, shadowing a professional, and interactive small group teaching. But also, shorter lectures on educational theory were mentioned.

## Discussion

### Medical students’ experiences of peer teaching

The medical students described positive experiences with PAL, including both PTs’ experiences of teaching and PLs’ preconceptions of possible beneficial outcomes. For example, in accordance with previous research [8], both PTs and PLs recognized increased subject knowledge as a major learning outcome of being a PT. In addition to the opportunity to repeat subject knowledge, the interviewed students became PTs for different reasons such as social, altruistic, financial, or professional, findings corresponding to similar results from studies on what motivates students to participate in voluntary teacher training programs [15].

The statements made by PTs indicate that the experience, also in line with previous research, leads to professional development such as professional behavior, communication skills, teamwork and teaching skills [8, 9, 16, 17]. However, these advantages were not as commonly seen in the PLs’ reflections, indicating that partaking in teaching may result in greater learning outcomes than expected by students when starting a PT-assignment. For example, PTs distinguished between repetition to retrieve knowledge and the act of teaching to strengthen their knowledge. Another result was that more experienced PTs realized their own limitations resulting in reflections on their strengths and weaknesses, an important skill for physicians who should be able to identify knowledge gaps and continually work to address these [1]. This change in attitude towards own learning and knowledge is supported by results from a previous study where PTs used PLs questions, helping PLs with difficult tasks, and evaluating judgement during dialogues with PLs [18]. Another benefit of adding peer teaching to the undergraduate medical curriculum, related to professionalism, is that PTs have been shown to be more likely to train for and include teaching during their future careers [17, 19], and participation in student-as-teacher courses may increase interest in medical education [20]. Students’ involvement in teaching in the future was not a research question in this study, however, it is an important aspect to consider when deciding to include PAL in the medical curriculum.

The results from this study, as well as previous research [21, 22], show that a majority of PTs receive little or no formal training before starting to teach. The existing training described by the PTs in this study included mostly information on practical issues and the opportunity to put questions to faculty or more experienced PTs. However, despite the lack of formal support, PTs manage to gain positive experiences from PAL and the interviewed students shared the perception of teaching as a skill required of their future profession.

### Medical students’ approaches to teaching

With the lack of a formal support system, PTs described several strategies to support their teaching role. For example, they used PLs’ feedback, teachers and peers as role models and their own experiences from being a student. In addition, both PTs and PLs described other teaching opportunities during medical school (e.g. mentorship, arranging formative OSCEs) as well as extracurricular activities (e.g. being a scout leader or soccer coach), that strengthened them as teachers. Similar results were shown in a study in the Netherlands showing that medical students gained experience in teaching during medical school but also outside the formal curriculum [21]. A drawback with informal training is the lack of integration with the curriculum, as well as the possibility to arrange support for and assessment of students. Also, it does not give equal opportunities for all students.

Students’ perceived self-efficacy has been shown to influence the activities they choose to participate in as well as their future participation and success in the workplace [23]. One barrier to becoming a PT expressed by students in this study was a lack of confidence. Hence, building students’ self-confidence needs to be supported by the curriculum. Peer teachers have been shown to have a greater confidence when teaching [24, 25] and this confidence lasts over time after graduation [17]. Furthermore, intervention with a training program has been shown to increase medical students’ confidence in a teaching role [26]. Additionally, medical students who received formal training and experience in teaching had higher confidence and better readiness to act as teaching physicians [20].

### Support needed according to students

When including PAL, PTs wanted clear guidelines surrounding teaching sessions. The fact that faculty members are not always present in the current system can create uncomfortable situations for PTs in situations regarding logistics or unprofessional behavior. A previous study showed similar results, where anatomy tutors saw a need for including the logistics surrounding dissection and how to handle student challenges in a training program [27]. Furthermore, when supervisors of PTs were asked to identify needs for support, they listed didactic principles as well as practical skills in communication and giving feedback [28]. The results of the present study show that it is likely that all PT training should involve a combination of didactic and practical, as well as subject specific parts.

Students also highlighted the importance of having an option on what subject area to teach, as this would increase motivation and result in better outcomes for the PLs. It was important to students in this study that training should be contextualized and useful for their future profession, and that it involved practice with feedback. One suggestion from students, as a way for senior students to prepare for teaching in a clinical setting, was that senior students should support junior peers in performing clinical skills. This method has been successful in OSCEs [29] and clinical skills training [30].

### How to design teacher training according to students

Although students felt that they had neither received training before their PT-assignment, nor received training as part of their studies in general, and despite the fact that students considered teaching skills a central part of the professional role, they had mixed opinions about making peer teaching a mandatory part of undergraduate medical education. One factor contributing to interviewees being hesitant was curriculum overload, an established challenge to implementing programs [26]. By using PAL as a way for students to gain practical teaching experience instead of creating a separate course in teaching, the time constraint might be limited to an extent. In addition, this will produce the well-known benefits of PAL to the students that are taught [8]. Our results show that it is important to students that time is allocated for PAL in the schedule, and as scheduling was identified as one of the perceived barriers to become a PT, it is important to consider time and logistics when making peer teaching a part of the curriculum.

Another major reason for not making PAL mandatory was a concern that unmotivated PTs would perform their task with poor quality. They suggested that including PAL later in the curriculum would increase motivation as students soon would be supervisors and would therefore see the need to train their teaching skills. Timing is important as the students are more likely to engage in the activity if they can see the meaning of it. For example, Burgess et al. showed that students that were currently involved in teaching entered a voluntary training program as they enjoyed teaching and saw the benefits of formal training [15]. It has also been suggested that PAL has a greater benefit as part of the medical curriculum for all students, not only those who are interested [12, 31]. We argue that as teaching is considered an essential skill for a future medical doctor, training should be mandatory for all students. To make it enjoyable and thus motivating for medical students, it is important to consider the barriers to PAL raised in this study. Motivation could be increased by allowing students to choose the area in which they teach, and hesitancy could be decreased by facilitating timing and logistics.

The quality of teaching by PTs could be improved by including formative feedback and assessment. This would also support learning for the students that teach. The literature on best practice assessment of students teaching other students is sparse [12] and should most likely include a combination of several aspects e.g. subject specific knowledge, teaching skills and professional behavior. As teaching skills are expected learning outcomes of a medical undergraduate curriculum, appropriate learning and assessment activities should be included. The lack of systematic training is not unique to this study; a systematic review found merely 19 student teacher training programs in health professions education, most in medical education, only two of these were part of the core curriculum [3]. Although worldwide, teacher training is sparse, half of the participants in a study answered that they feel prepared to teach or mentor students [22]. We argue that by including mandatory teacher training in the medical curriculum, this number would increase, and the quality of teaching would improve.

## Methodological considerations and implications for practice

Our study provides a nuanced perspective on medical students’ experiences of peer teaching. Whilst the study was conducted at one institution only, data was gathered from students in both teacher and learner roles, from various peer assisted learning activities and with a range in regards of how much time had passed since the PT/PL experiences. The interviews were conducted by a medical student rather than a faculty member which is likely to have led to sincere responses aligning with the concept of social and cognitive congruence according to Loda et al. [5]. The different perspectives provided by the research team strengthened the quality of the study and the credibility is further strengthened by the identification of links to previous research in the field.

Further research to explore includes evaluation of implemented mandatory teacher training on a programmatic level as well as analysis of graduated medical students’ abilities to teach, supervise and perform their clinical duties. Possible analyses that can be made are effects on graduates’ interactions with patients and relatives, skills in supervision of junior students, and self-confidence in the teaching task.

## Conclusions

This study indicates that PTs develop subject specific knowledge and professional skills such as teaching and communication skills. However, barriers were identified for some students to take on the role as PT, one of them being lack of confidence. At our institution there is lack of formal training for medical students in general during undergraduate education to be prepared for the educational assignment as doctors. Such training could help address some of the perceived barriers for taking on a teaching role. The results from this investigation highlight students’ perceptions of the content and format of a peer teaching program if it was to be implemented for all medical students. Students want to learn through practical experience with feedback and in an appropriate setting mirroring the educational assignment of the future profession which is important to consider when implementing peer teaching in future medical curricula.

## Electronic supplementary material

Below is the link to the electronic supplementary material.

Supplementary Material 1

## Data Availability

Data cannot be shared publicly because the data contain potentially identifying study participants’ information. Some data might be available upon request from Karolinska Institutet (contact via ellinor.kenne@ki.se) for researchers who meet the criteria for access to confidential data (please provide the manuscript title with your enquiry).

## Abbreviations

KI: Karolinska Institutet
PAL: Peer assisted learning
PL: Peer learner
PLs: Peer learners
PT: Peer teacher
PTs: Peer teachers
