# Supplementary material for: Learning to teach by teaching your peers: exploring students’ needs for training in the undergraduate medical education curriculum
Source: BMC Med Educ. 2025 Mar 21;25:414. doi: 10.1186/s12909-025-07022-z (PMC11927351; doi:10.1186/s12909-025-07022-z)
Supplement: Supplementary file 1 — Supplementary Material 1 [file 12909_2025_7022_MOESM1_ESM.pdf]

Interview guide

Questions for peer teachers (focus group interview)

1. Why have you chosen to teach?
2. What expectations did you have? What was it like to teach? Did anything surprise you? If not active in teaching anymore: Why did you quit? Would you have done anything differently if you did it again?
3. What education/training have you received from the employer? If you received training, was it particularly useful?
4. How do you cooperate with the course manager/teacher at the department? What type of assignment have you been given? How bound or free are you in your approach to teaching? Why did you choose your setup for teaching? Did you feel that the structure of the teaching worked well (reactions from students)?
5. Have you missed any type of training for the teaching assignment you have had? (Both regarding subject knowledge and pedagogy). If yes, how do you think such education/training would best be designed?
6. Is there a need for further training after starting an assignment? If so, how would it best be designed?
7. Did you receive any feedback on your work? In what form was the feedback?
8. In what way has teaching influenced your professional development? In what way can your experiences be useful in your future profession as a doctor?
9. How has peer teaching affected your own learning?
10. What have you learned? Can you share something you have learned from teaching others?
11. What do you think is your most important function as a peer teacher?
12. For whom is Peer-assisted learning best, the student or the teacher?
13. Are there other ways in which peer-learning could be used in the medical program?
14. Should it be mandatory for students in the medical program to teach other students/patients? Why/why not? If no; Should pedagogical training be compulsory in the medical program?
15. Is there a question that was not included that you think I should have asked?
16. How did you experience this focus group interview?

Questions for peer learners (focus group interview)

1. Based on your experiences, what advantages/disadvantages do you see with peer teaching?
2. Why have you chosen to not to teach?
3. Should it be mandatory for students in the medical program to teach other students? Why/why not?
4. If teaching were to be a mandatory part of the medical program, what type of training/education would you like to receive?
5. Is there anything that medical program could do to make you as a student want to teach other students?
6. Is there a question that was not included that you think I should have asked?
7. How did you experience this focus group interview?

Questions for newly examined doctors that have been peer teachers (semistructured individual interview)

1. Describe your teaching assignment
   a. How long did you teach (at KI's medical program)?
   b. What duties did you have?
   c. What extent (hours/weeks/semesters)?
2. What education/training did you receive from KI?
   a. Was it particularly useful?
3. Has any other teaching in the medical program helped you (besides subject knowledge in the area you taught)? If yes, describe what.
4. Have you missed any type of training for the assignment you have had? Both regarding subject knowledge and pedagogical training?
   a. How do you think such a system would best be designed?
   b. Is there a need for further training after you started an assignment? If so, how would it best be designed?
5. Why did you choose to teach?
6. Should it be mandatory for students in the medical program to teach other students? Why/why not? If no, should the medical program include pedagogical training?
7. Does your current work include any teaching assignment (incl. supervision)?
   a. Did you choose that yourself?
   b. Is your education/experience from the medical program sufficient to carry out the assignment in a good way?
   c. Have you had help from teaching during your undergraduate education?
8. Is there a question that was not included that you think I should have asked?
9. How did you experience this interview?

Questions for newly examined doctors that have <u>not</u> been peer teachers (semistructured individual interview)

1. Based on your experiences, what advantages/disadvantages do you see with peer teaching?
2. Why have you chosen to not to teach during your studies at KI?
3. Should it be mandatory for students in the medical program to teach other students? Why/why not? If no, should the medical program include pedagogical training?
4. If teaching were to be a mandatory part of the medical program, what type of training/education would you like to receive?
5. Does your current work include any teaching assignment (incl. supervision)?
   a. Did you choose that yourself?
   b. Is your education/experience from the medical program sufficient to carry out the assignment in a good way?
6. Is there a question that was not included that you think I should have asked?
7. How did you experience this interview?
